# Ultrasound-guided transfection of claudin-5 improves lung endothelial barrier function in lung injury without impairing innate immunity

**DOI:** 10.1152/ajplung.00107.2023

**Published:** 2023-06-13

**Authors:** Rajiv Sanwal, Victoria Mintsopoulos, Mihails Ditmans, Alice Lang, Elyse Latreille, Siavash Ghaffari, Negar Khosraviani, Raffi Karshafian, Howard Leong-Poi, David M. Hwang, Laurent Brochard, Alberto Goffi, Arthur S. Slutsky, Warren L. Lee

**Affiliations:** ^1^Department of Laboratory Medicine and Pathobiology, University of Toronto, Toronto, Ontario, Canada; ^2^Keenan Research Center for Biomedical Science, St. Michael’s Hospital, Toronto, Ontario, Canada; ^3^Department of Physics, Toronto Metropolitan University, Toronto, Ontario, Canada; ^4^Institute for Biomedical Engineering, Science and Technology (iBEST), Toronto, Ontario, Canada; ^5^Department of Laboratory Medicine and Molecular Diagnostics, Sunnybrook Health Sciences Centre, Toronto, Ontario, Canada; ^6^Interdepartmental Division of Critical Care Medicine, University of Toronto, Toronto, Ontario, Canada

**Keywords:** claudin-5, endothelial barrier, lung injury, transfection, vascular leakage

## Abstract

In acute lung injury, the lung endothelial barrier is compromised. Loss of endothelial barrier integrity occurs in association with decreased levels of the tight junction protein claudin-5. Restoration of their levels by gene transfection may improve the vascular barrier, but how to limit transfection solely to regions of the lung that are injured is unknown. We hypothesized that thoracic ultrasound in combination with intravenous microbubbles (USMBs) could be used to achieve regional gene transfection in injured lung regions and improve endothelial barrier function. Since air blocks ultrasound energy, insonation of the lung is only achieved in areas of lung injury (edema and atelectasis); healthy lung is spared. Cavitation of the microbubbles achieves local tissue transfection. Here we demonstrate successful USMB-mediated gene transfection in the injured lungs of mice. After thoracic insonation, transfection was confined to the lung and only occurred in the setting of injured (but not healthy) lung. In a mouse model of acute lung injury, we observed downregulation of endogenous claudin-5 and an acute improvement in lung vascular leakage and in oxygenation after claudin-5 overexpression by transfection. The improvement occurred without any impairment of the immune response as measured by pathogen clearance, alveolar cytokines, and lung histology. In conclusion, USMB-mediated transfection targets injured lung regions and is a novel approach to the treatment of lung injury.

**NEW & NOTEWORTHY** Acute respiratory distress syndrome is characterized by spatial heterogeneity, with severely injured lung regions adjacent to relatively normal areas. This makes targeting treatment to the injured regions difficult. Here we use thoracic ultrasound and intravenous microbubbles (USMBs) to direct gene transfection specifically to injured lung regions. Transfection of the tight junction protein claudin-5 improved oxygenation and decreased vascular leakage without impairing innate immunity. These findings suggest that USMB is a novel treatment for ARDS.

## Graphical abstract

**Figure:**
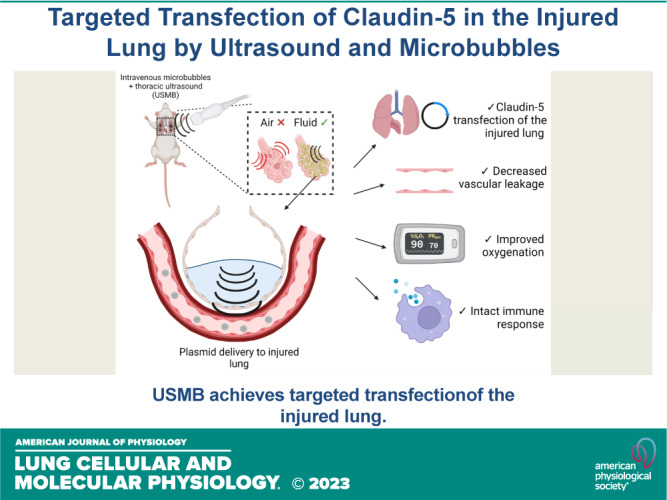

## INTRODUCTION

Acute lung injury is a common diagnosis in critical care units and in its most severe form has a mortality rate approaching 40% (1). Common causes include pathogen-induced pneumonia (2) as well as aspiration or inhalational injury. It is characterized by increased permeability of the alveolar-capillary membrane (3), resulting in alveolar flooding and hypoxemic respiratory failure. Other than management of the underlying cause (e.g., antimicrobial therapy in the setting of an infection), there is currently no specific treatment; patients usually require mechanical ventilation to stay alive until the lung heals itself.

Regardless of the cause, most forms of acute lung injury demonstrate heterogeneous involvement of the lungs within a given patient: densely consolidated, edematous, or atelectatic lung is observed adjacent to other lung regions that appear relatively normal (4). This heterogeneity makes it difficult to target therapy to the most injured areas. Mechanical ventilation, while life-saving, preferentially distributes to the more compliant areas, predisposing them to overdistension and further lung damage (5). Drugs administered by inhalation similarly distribute to the compliant regions, increasing the risk of off-target effects. The administration of therapies via a systemic route is also suboptimal as other organs will be affected.

Regionally targeted treatment delivered early may be important to prevent further extension with clinical deterioration. We hypothesized that the loss of air in the injured lung could be leveraged to enable targeted and increased delivery of drugs and genetic material using ultrasound and microbubbles (6). Microbubbles are composed of a lipid outer shell that encapsulates an inert gas; numerous formulations are already used clinically as echocardiographic contrast agents, including in critically ill patients (7). When exposed to ultrasound energy (i.e., insonated), microbubbles oscillate and cavitate and this physical reaction stimulates enhanced endocytosis and transient sonoporation at the plasma membrane of surrounding cells (8). This reaction can be harnessed to induce delivery of cargo bound to or vicinal to the microbubbles. Although studied in other fields (9, 10), this effect has received scant attention in the lung because ultrasound energy is readily blocked by air.

We reasoned that in acute lung injury, ultrasound waves will preferentially penetrate the most damaged areas of the lung, i.e., fluid-filled, or atelectatic regions, leaving more normal (air-filled) areas of the lung unaffected. Ultrasound energy that penetrates severely injured lung regions could then act on circulating microbubbles, enhancing local gene or drug delivery. Since the ultrasound transducer can be focused on the thorax, the effect on other organs is limited, further increasing the specificity of delivery.

In this report, we establish the feasibility of this approach (termed USMB) for the delivery of genetic cargo to the injured lungs of animals. We find that USMB can achieve transfection in a highly spatially restricted manner, resulting in functional protein expression in both lung endothelial and epithelial cells only in injured and insonated lungs. We show that USMB-mediated transfection of the tight junction constituent claudin-5 causes decreased vascular leakage and a significant improvement in oxygen saturation in mice with lung injury. This improvement occurs without impairing innate immunity or pathogen clearance. Together, these data indicate that USMB-mediated gene delivery is feasible as a targeted therapeutic approach for acute lung injury.

## METHODS

### Animals

All experimental procedures were approved by the St. Michael’s Hospital Animal Care Committee (ACC 772/120, 882). C57BL/6J male mice (10–14 wk old) were purchased from Jackson Laboratories; in pilot studies, we observed the efficacy of USMB-mediated transfection in animals of both sexes. Only male mice were used in this study to limit costs.

### Treatment with Ultrasound and Microbubbles

Unless otherwise indicated, a SONOS 5500 (Phillips) ultrasound machine and an S3 phased array transducer were used. The following ultrasound settings were used: transmitter frequency, 1.3 MHz; 67 V; 0.2 W; mechanical index, 0.9; pulse interval, 5 s. Cationic microbubbles for plasmid transfection were prepared as previously reported (9). Microbubble concentrations, charge, and size (200–300 nm) were determined using a Coulter counter (Beckman-Coulter) and DLS Zetasizer Ultra (Malvern Pananalytical) before each experiment.

For *E. coli*, mice were intratracheally infected with 6 × 10^7^ colony-forming units (CFUs) of *E. coli* (OD = 1.0) in a volume of 130 μL; healthy controls received no fluid. Furthermore, 0.5–6 h postinfection (hpi), mice were intravenously administered 10 μg plasmid with 3 × 10^8^ cationic microbubbles in a volume of 300 μL followed immediately by 5 min of ultrasound on each (left and right) side of the chest. Sixteen to twenty-four hours posttreatment, mice were euthanized via cardiac puncture. For luciferase experiments, 24-h post-USMB mice were injected intraperitoneally with 2.25 mg of d-luciferin dissolved in 150 μL of PBS. After 15 min, mice were euthanized and harvested for lung and kidney. Organ luminescence was detected using a Newton 7.0 FT-500 Bioluminescent and Fluorescent Animal Imager. Luminescent signal was analyzed by subtracting background luminescence of control animal organs that did not receive luciferin.

**Figure 1.**
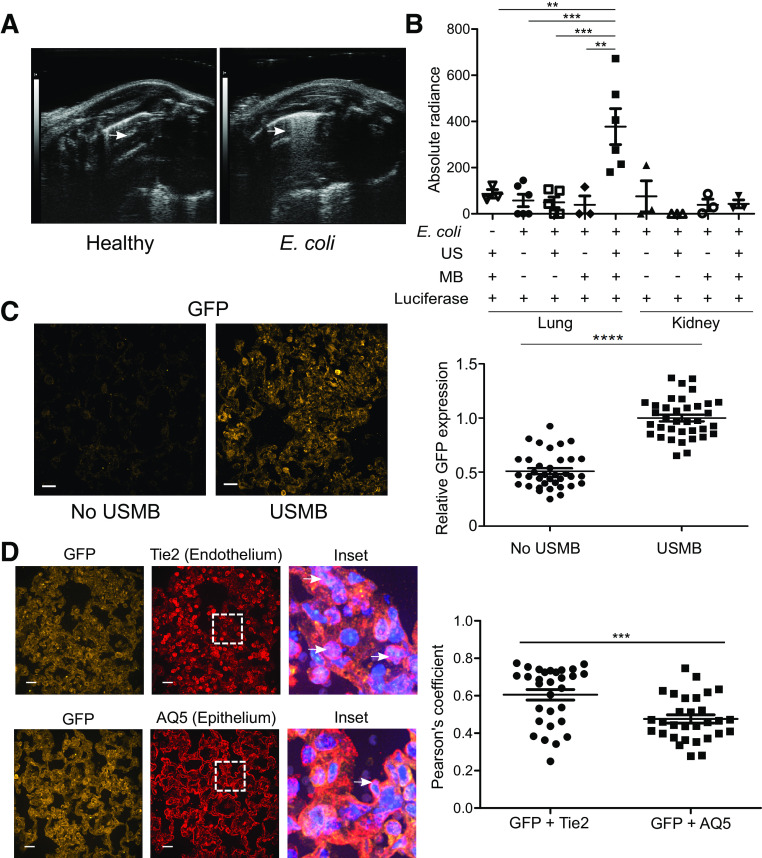
Ultrasound-microbubble (USMB)-mediated transfection achieves functional gene delivery in a murine *Escherichia coli* (*E. coli*) pneumonia model. *A*: representative lung ultrasound image from mice infected intratracheally (IT) with *E. coli* demonstrating characteristic B lines (*right*, white arrow) compared with the A lines of healthy controls (*left*, white arrow); images were acquired using a Vevo 2100 Imaging system (VisualSonics). *B*: USMB-mediated transfection of Firefly-luciferase plasmid to the injured lung followed 24 h later by injection of luciferin results in lung luminescence that requires the combination of ultrasound (US), microbubbles (MB), and lung injury. Mice were infected with *E. coli* and treated 2 h post infection; ***P < 0.001, **P < 0.01, each point represents one animal. *C*: USMB-mediated transfection of a plasmid encoding green fluorescent protein (GFP) in *E. coli-*infected mice. Immunofluorescent staining demonstrates significantly increased GFP expression in treated animals compared with untreated controls. Scale bars = 15 µm; ****P < 0.0001, each point represents one field. *D*: USMB-mediated GFP transfection of the injured lung occurs to a greater extent in the endothelium than the epithelium. Images show staining for GFP and endothelium (Tie2) or epithelium (aquaporin 5, AQ5); colocalization was measured by Pearson’s coefficient; ***P < 0.001. Scale bar = 15 µm; each point represents one field. Error bars represent standard error of the mean.

For Evans Blue measurements, mice received thoracic ultrasound using the Vevo 2100 Imaging system (VisualSonics) with the MS250 transducer with burst mode activated every 6 s to achieve cavitation. Sixteen hours later mice were injected intravenously with 200 µL of 0.5% Evans Blue in PBS. After 1 h, mice were anesthetized and euthanized via cardiac puncture. Blood, lungs, and bronchoalveolar lavage fluid (BAL) were collected as previously described (11). Evans Blue was extracted and measured via spectroscopy as previously described (12). To obtain images of edematous and healthy mouse lungs (e.g., Fig. 1*A*), the Visual Sonics Vevo 2100 Imaging System with the MS400 probe was used.

For influenza, mice were intranasally infected with 2 × 10^5^–1.5 × 10^6^ plaque-forming units (PFUs) of influenza A (HKx31;H3N2, from Dr. Tania Watts) and diluted to a final volume of 130 µL as previously described (13). To mimic a clinical scenario, animals received oseltamivir phosphate (Sigma-Aldrich) (10 mg/kg, ip) twice daily starting 48 h after infection for a total of 5 days.

The Mouse Ox Plus device and software (Starr Life Sciences, Oakmont, PA) were used to measure arterial oxygen saturation of infected mice immediately before and 24 h after USMB treatment. Prior to infection, neck hair was shaved so that the small collar could accurately obtain the ${\text{S}}_{{\text{P}}_{{\text{O}}_{2}}}$ measurement; measurements were made daily but regrowth of hair and weight loss precluded accurate measurements more than 6 days after infection. Body weight, temperature, and activity score [(scored from 1 (moribund) to 4 (normal), as previously described (13)], were measured daily; mice were euthanized if two or more of the following occurred: weight loss exceeded 30% of initial weight; temperature fell below 31°C; oxygen saturation below 75%, or the animal appeared moribund (activity score of 1). Cytokine profiles were measured using the V-PLEX Cytokine Panel 1 Mouse Kit and V-PLEX Plus Proinflammatory Panel 1 Mouse Kit (MSD, K15245G, and K15048G).

For histological analysis, lungs were fixed for 48 h using 10% neutral-buffered formalin and embedded in paraffin. Furthermore, 5-μm thick slides were prepared. For immunofluorescent staining, we used anti-Tie-2 (Santa Cruz Biotechnology, H-176, sc-9026), anti-aquaporin-5 (Abcam, ab78486), and anti-GFP. For hematoxylin and eosin staining, the Leica Autostainer XL was used. Slides were analyzed in a blinded fashion according to consensus criteria (14).

### qPCR

For qPCR analysis, RNA was extracted from whole lung lysates using TRIzol. cDNA was generated using PowerUp SYBR Green Master Mix (Applied Biosystems). Primers used (5′ to 3′) were: human claudin-5 forward GGATCACTCTCGGCATGGAC, human claudin-5 reverse CCTCTTTGAAGGTTCGGGGG, mouse 18 s forward GTAACCCGTTGAACCCCATT, and mouse 18 s reverse CCATCCAATCGGTAGTAGCG.

### Cell Culture

Primary human pulmonary microvascular endothelial cells (PromoCell C-12281) were grown on transwells (Costar, 3460; for transendothelial electrical resistance measurements, TEER) or glass coverslips (for immunofluorescent imaging). TEER was measured using Endohm-12 from World Precision Instruments. For imaging, cells were fixed with 4% PFA and counterstained with DAPI. Confocal imaging was performed with a Quorum TIRF/SD microscope.

### Statistical Analysis

All experiments were repeated a minimum of three times independently; data are presented as means and standard errors of the mean. In experiments with two groups, the *P* value was determined by two-tailed *t* tests. In experiments with more than two groups, data were analyzed by one-way ANOVA and post hoc *t* tests when appropriate. For the change in oxygen saturation 24 h after USMB, a one-way ANOVA with mixed-effects analysis was used due to unequal sample sizes. Statistical comparisons were completed using GraphPad Prism software (La Jolla, CA).

## RESULTS

### Ultrasound and Intravenous Microbubbles Achieve In Vivo Transfection of Injured Lung

We infected C57BL/6 mice intratracheally with *E. coli*, a common cause of nosocomial pneumonia. Mice rapidly develop hypoxemia, pulmonary edema, and alveolar neutrophilia (15), typical of acute lung injury (4). Thoracic ultrasound of *E. coli*-infected mice revealed B lines characteristic of pulmonary edema (16), compared with the expected A lines observed in healthy animals (Fig. 1*A*). Using this model, we then injected the mice intravenously with cationic microbubbles bound to a plasmid encoding the enzyme luciferase; this was followed by administration of ultrasound to the chest bilaterally for 5 min using a standard clinical transducer. Twenty-four hours later, mice were injected intraperitoneally with the enzymatic substrate, luciferin. Lungs were excised 15 min later from euthanized animals and the lungs’ luminescence was measured. We observed a significant increase in light in the lungs of USMB-luciferase-treated animals (Fig. 1*B*). Light was absent from the kidneys of the same animals, indicating that transfection was not generalized. The results indicated that the generation of light required the simultaneous presence of injured lung, thoracic ultrasound, intravenous microbubbles, and the luciferase plasmid.

To determine which cells in the injured lung are transfected by this method, we used USMB to deliver a plasmid encoding a green fluorescent protein (GFP); 24 h later, lungs were analyzed by immunofluorescence. We observed GFP protein in treated but not control animals (Fig. 1*C*) as well as significant colocalization of GFP with Tie2 (an endothelial marker) and aquaporin-5 (an epithelial marker) (Fig. 1*D*). GFP colocalized with the lung endothelium to a significantly greater degree than the epithelium. Together, these data indicate that USMB achieves gene transfection and functional protein expression in vivo in the injured lung.

### Transfection of Claudin-5 Decreases Lung Vascular Leakage In Vivo

**Figure 2.**
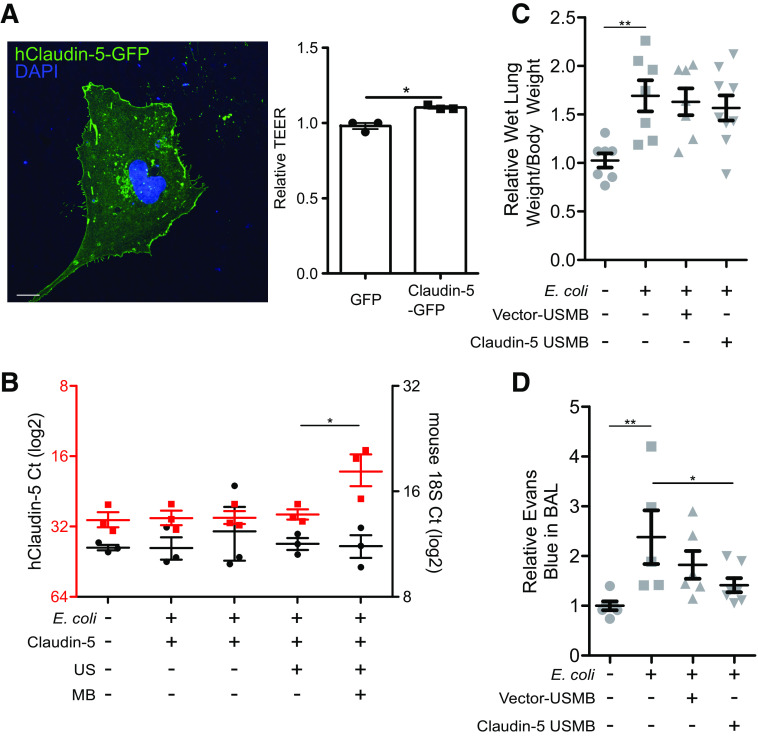
Claudin-5 levels regulate barrier function in cultured endothelial cells and in the injured lung. *A*: transfection of cultured human pulmonary microvascular endothelial cells with claudin-5-GFP indicates appropriate targeting of the protein to the plasma membrane (*left*). Scale bar = 9 µm. Transfection results in increased barrier function as shown by transendothelial electrical resistance compared with empty vector (TEER) (*right*; *P < 0.05; each point is an independent experiment). *B*: ultrasound-microbubble (USMB)-mediated transfection of claudin-5 achieves gene delivery to the injured lung. Mice were infected with *E. coli* IT and treated with USMB 30 min postinfection. Sixteen hours post-USMB, lungs were collected and homogenates were analyzed by qPCR; *y*-axis is cycle number in log2; *P < 0.05, each point represents one animal. *C* and *D*: sixteen hours post-USMB, Evans Blue dye was injected retro-orbitally and tissues were collected 1 h after injection. *C*: the ratio of wet lung weight to total body weight is shown; *P < 0.01, each point represents one animal. *D*: Evans Blue leakage into BAL was significantly reduced by claudin-5-USMB; **P < 0.01 and *P < 0.05, each point represents one animal. GFP, green fluorescent protein; IT, intratracheally.

Given that vascular leakage to plasma is not required for leukocyte emigration from the circulation during inflammation (17), we reasoned that enhancing vascular integrity might constitute a viable therapeutic strategy for lung injury by decreasing pulmonary edema and improving oxygenation. We hypothesized that USMB could be used to deliver barrier-stabilizing genetic material to the injured lung. We chose the tight junction constituent claudin-5, a transmembrane protein that is enriched in endothelial cells and is expressed endogenously at high levels in the lung and brain. Mice deficient in claudin-5 exhibit an increase in vascular leakage (18). Transfection of cultured human lung endothelial cells with claudin-5 significantly increased barrier function in vitro as measured by transendothelial electrical resistance (TEER); we also confirmed that the transfected protein is targeted appropriately to the plasma membrane in cells (Fig. 2*A*).

We next confirmed USMB transfection of human claudin-5 using the *E. coli* model of lung injury; using a plasmid for human claudin-5 allowed us to design species-specific PCR primers to distinguish the transfected plasmid from the endogenous transcript. Sixteen hours after treatment, we observed significantly increased expression of human claudin-5 in USMB-treated animals (Fig. 2*B*); in contrast, murine claudin-5 levels were unchanged or slightly lower than in healthy animals (data not shown). Transfection caused a trend toward decreased lung edema that was not statistically significant (Fig. 2*C*); nonetheless, we observed a significant reduction in lung vascular leakage when measured directly by Evans Blue extravasation into bronchoalveolar lavage fluid (BAL) (Fig. 2*D*). However, as mice recover spontaneously and rapidly from this infection, it was not possible to detect any difference in oxygen saturation or survival between treated and untreated animals (data not shown).

### Transfection of Claudin-5 Improves Oxygenation in Influenza-Induced Lung Injury

**Figure 3.**
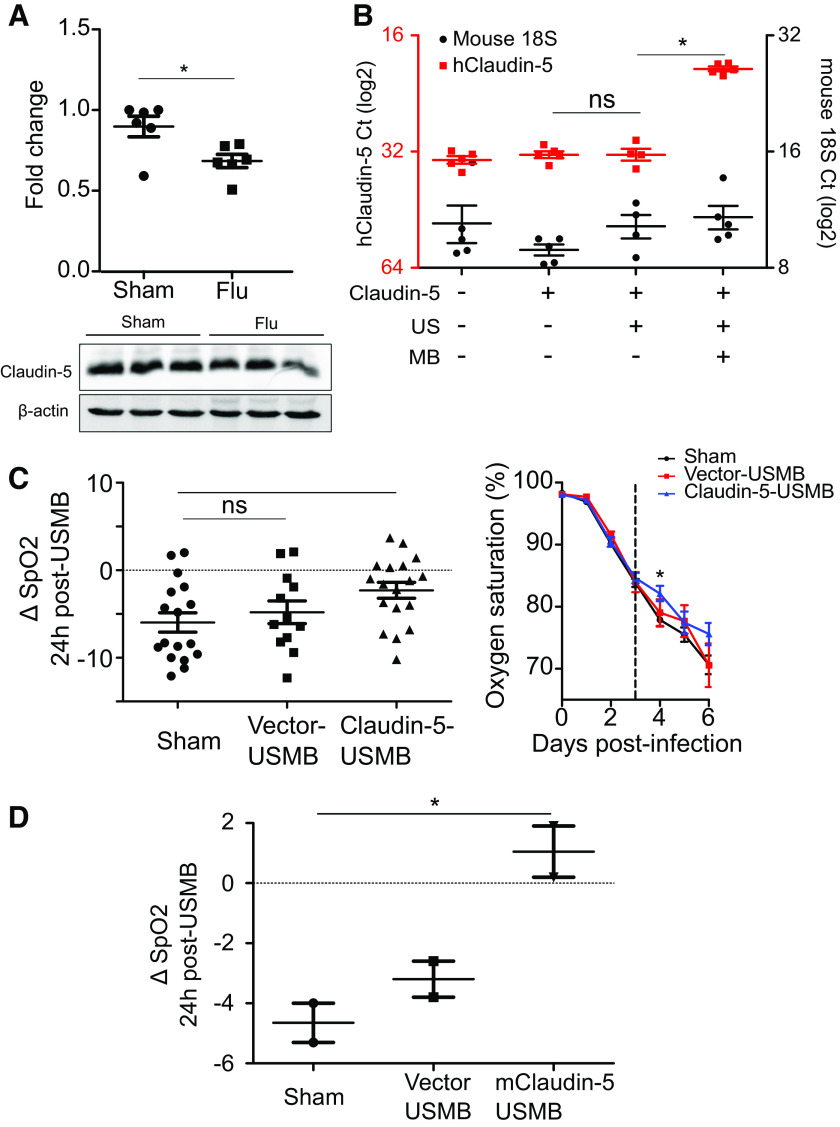
Claudin-5 transfection improves oxygenation in influenza-induced lung injury. *A*: endogenous claudin-5 protein is downregulated in influenza-infected mouse lungs 3 days postinfection (*P < 0.05, each point represents one animal). Representative Western blot of lung homogenates from separate animals is shown. *B*: successful gene delivery 24 h after ultrasound-microbubble (USMB)-mediated transfection of claudin-5 to the lung as measured by qPCR; *y*-axis is cycle number in log2; *P < 0.05, each point represents one animal. *C*: transfection of claudin-5 results in significant improvement in oxygenation 24 h postdelivery compared with controls. Left graph shows oxygen saturation of all groups over time with hatched vertical line indicating the day of treatment. Right scatter plot shows the change in oxygen saturation (${\text{S}}_{{\text{P}}_{{\text{O}}_{2}}}$) 24 h after USMB; *P < 0.05; each point represents one animal. *D*: USMB-mediated transfection of murine claudin-5 (mClaudin-5) results in improved oxygenation 24 h after treatment; vector-alone control group is the same as in *C*; *P < 0.05, each point represents one animal.

To better detect any physiological impact of transfection, we used an influenza-induced model of lung injury. Mice are infected intranasally with influenza A resulting in the development of alveolar neutrophilia, hypoxemia, and lung edema (13, 19, 20). We have previously reported that claudin-5 expression is decreased in cultured lung endothelial cells exposed to human influenza (21); here we found that mice infected with influenza also show decreased expression of claudin-5 in lung homogenates (Fig. 3*A*). Three days after infection with influenza A, mice were injected intravenously with microbubbles bound to a plasmid encoding human claudin-5 followed immediately by treatment with thoracic ultrasound. Twenty-four hours later, we observed significant expression of human claudin-5 (Fig. 3*B*) and a significant improvement in oxygenation relative to control animals (Fig. 3*C*). To account for species-specific differences, we repeated the experiment using a plasmid encoding murine claudin-5 and observed a similar benefit in terms of oxygenation 24 h later (Fig. 3*D*).

### Enhancing the Lung Endothelial Barrier Does Not Compromise Innate Immunity

**Figure 4.**
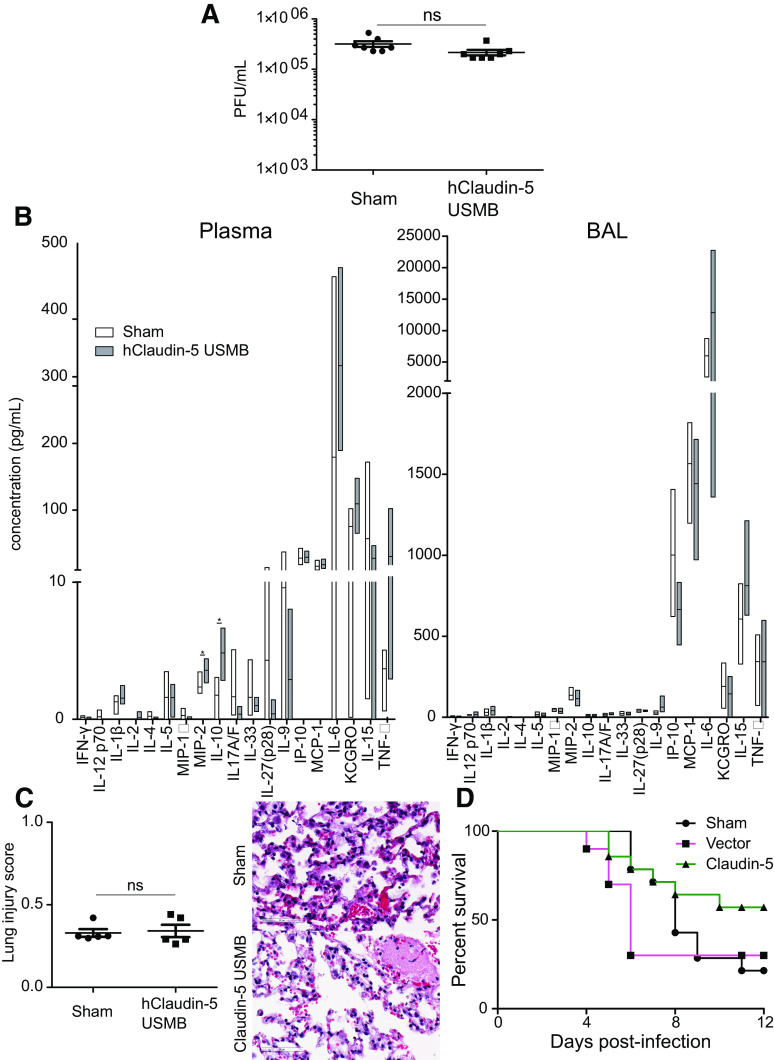
Transfection of claudin-5 does not compromise innate immunity. *A*: viral titer in lung homogenates does not differ between treated and untreated animals as measured by plaque assay 24 hours after treatment *B*: plasma and bronchoalveolar lavage (BAL) cytokine profiles are comparable between the two groups (data shown as mean with minimum to maximum) as are (*C*) lung injury scores of histological sections. Scale bars = 60 µm. *D*: survival was not significantly different between treated animals and controls.

We wanted to verify that the improved oxygenation did not come at the expense of an appropriate immune response to the virus. Transfection of claudin-5 had no effect on pathogen clearance, as viral titers in lung homogenates were unchanged (Fig. 4*A*). Cytokine levels in bronchoalveolar lavage fluid were also unchanged between groups (Fig. 4*B*). Similarly, histological scoring of lung injury by a lung pathologist blinded to groups was identical, indicating that neutrophil recruitment to the lung was unimpaired (Fig. 4*C*). Although it appeared higher, the survival rate in USMB-claudin-5 treated animals was not statistically different from controls (Fig. 4*D*; *P* = 0.12).

## DISCUSSION

Acute lung injury is a common complication of infection with many viral and bacterial lung pathogens, including influenza A and SARS-CoV-2 (22). It is also a common consequence of aspiration or inhalational injury. In both cases, there is no specific therapy. Spatial heterogeneity of lung involvement in individual patients constitutes an additional hurdle that predisposes to ventilator-induced lung injury and other off-target effects. Even full acute respiratory distress syndrome (ARDS) is characterized by spatial heterogeneity that renders therapy risky for the less injured lung or inefficient for the atelectatic lung (4).

Although ultrasound and microbubbles have been used to enhance drug delivery in other organs (9), the reflection of ultrasound energy by air has severely limited its use in the lung. We have previously shown that USMB enhances lung deposition of an aminoglycoside antibiotic and increases bacterial killing in a mouse model of pneumonia-induced lung injury (6), findings confirmed by others (23). Here we demonstrate for the first time, to our knowledge, that the combination of thoracic ultrasound and intravenous microbubbles (USMBs) confers the ability to transfect the injured lung and to improve lung physiology. We show that USMB-mediated genetic delivery is specific to the injured lung and requires direct insonation by ultrasound, making it highly spatially targeted. This holds theoretical advantages over other methods of in vivo transfection in the lung, such as nanoparticles or viral vectors. Such approaches tend to distribute preferentially to organs outside of the thorax (e.g., the liver) or to normal areas of the lung, potentially leading to off-target effects. In contrast, the requirement for simultaneous and colocalized thoracic ultrasound, circulating microbubbles, and injured lung confers exquisite temporal and spatial control to USMB-mediated transfection.

Our data indicating that transfection of claudin-5 is beneficial demonstrates that the overall strategy of USMB-mediated gene delivery has merit. Preventing vascular leakage regionally may prevent an extension of injury, provided that it does not prevent the action of local immunity. The pathognomonic abnormality in acute lung injury (and ARDS) is increased alveolar-capillary permeability and overexpression of claudin-5 addresses this directly. In vivo transfection using USMB did not aggravate lung injury as measured histologically, nor did it interfere with pathogen clearance or the generation of cytokine response in the lung. Instead, overexpression of claudin-5 in the injured lung significantly improved oxygenation and decreased vascular leakage; these data suggest that tightening of the vascular barrier by molecular means is feasible and does not necessarily compromise the immune response (17). More generally, our findings raise the possibility that USMB-mediated delivery of other genetic cargoes could confer similar or potentially even greater benefits in lung injury.

In this study, we have used plasmids given their ease of production in large quantities and the potential for their enhancement using customized (e.g., tissue-specific) promoters. The delivery of oligonucleotides such as miRNA and siRNA should also be feasible (9).

Despite our data in mice showing physiological improvement and no worsening of histological injury, USMB in patients with ARDS would require further study to ensure no acute adverse effects (hypotension, hypercoagulability, pulmonary hypertension, etc.). Sufficient tissue penetration of ultrasound may also be an issue in larger animals, although endobronchial ultrasound could in principle permit access to deeper areas of the injured lung. Optimization of USMB for its use in humans and delineation of its potential additional applications is now underway (24).

## GRANTS

This study was supported by Collaborative Health Research Program grant from the Canadian Institutes of Health Research (CIHR, CPG158284) and the Natural Sciences and Engineering Research Council of Canada (523598-18); Operating grant from the CIHR (OV2 170656); Canada Research Chair Program.

## DISCLOSURES

W.L.L., H.L.P., and R.K. are coinventors on a patent related to this work. W.L.L. is the Chief Scientific Officer and R.K. is the Secretary of a spin-off company related to this work. None of the other authors has any conflicts of interest, financial or otherwise, to disclose.

## AUTHOR CONTRIBUTIONS

R.S., V.M., R.K., H.L.-P., D.M.H., A.G., and W.L.L. conceived and designed research; R.S., V.M., M.D., A.L., E.L., S.G., and N.K. performed experiments; R.S., V.M., S.G., N.K., D.M.H., A.G., and W.L.L. analyzed data; R.S., D.M.H., A.G., and W.L.L. interpreted results of experiments; R.S. prepared figures; R.S. and W.L.L. drafted manuscript; R.S., L.B., A.G., A.S.S., and W.L.L. edited and revised manuscript; R.S., R.K., H.L.-P., D.M.H., L.B., A.G., A.S.S., and W.L.L. approved final version of manuscript.
